# Monitoring of Hidden Corrosion Growth in Aircraft Structures Based on D-Sight Inspections and Image Processing

**DOI:** 10.3390/s22197616

**Published:** 2022-10-08

**Authors:** Andrzej Katunin, Marko Nagode, Simon Oman, Adam Cholewa, Krzysztof Dragan

**Affiliations:** 1 Department of Fundamentals of Machinery Design, Faculty of Mechanical Engineering, Silesian University of Technology, Konarskiego 18A, 44-100 Gliwice, Poland; 2 Faculty of Mechanical Engineering, University of Ljubljana, Aškerčeva 6, SI-1000 Ljubljana, Slovenia; 3 Division of Airworthiness, Air Force Institute of Technology, Ks. Bolesława 6, 01-494 Warsaw, Poland

**Keywords:** corrosion, non-destructive testing, D-Sight, condition monitoring, image processing

## Abstract

Hidden corrosion in aircraft structures, not detected on time, can have a significant influence on aircraft structural integrity and lead to catastrophic consequences. According to the widely accepted damage tolerance philosophy, non-destructive inspections are performed to assess structural safety and reliability. One of the inspection techniques used for such an inspection is the optical D-Sight technique. Since D-Sight is used primarily as a qualitative method, it is difficult to assess the evolution of a structural condition simply by comparing the inspection results. In the following study, the method to monitor hidden corrosion growth is proposed on the basis of historical data from D-Sight inspections. The method is based on geometric transforms and segmentation techniques to remove the influence of measurement conditions, such as the angle of observation or illumination, and to compare corroded regions for a sequence of D-Sight images acquired during historical inspections. The analysis of the proposed method was performed on the sequences of D-Sight images acquired from inspections of Polish military aircraft in the period from 2002 to 2017. The proposed method represents an effective tool for monitoring hidden corrosion growth in metallic aircraft structures based on a sequence of D-Sight images.

## 1. Introduction

Corrosion in metallic aircraft frames and other aircraft elements is one of the most serious factors that influence structural integrity and safety. Together with fatigue, corrosion is the most costly type of damage that affects aircraft structures. According to various sources [1,2,3], the annual cost of corrosion in the aircraft industry is estimated to be several billion to several tens of billions of US dollars. This cost includes corrosion maintenance, inspection costs, aircraft downtime, etc. Furthermore, as reported in [4,5], corrosion is one of the leading causes of 10% to 25% of structural failures of aircrafts and is one of the leading causes of failure based on the number of aircraft accidents, which makes this problem significant and still current. With a continuous extension of the operational life of aircrafts, the problem of corrosion is receiving more and more attention.

In general, corrosion can be characterized as a complex interaction of chemical, mechanical, metallurgical, and microbiological processes [6,7], which in many cases act simultaneously. This, in turn, has a significant influence on other destructive processes, such as general fatigue, including several special types, such as stress corrosion, cracking, or hydrogen embrittlement [8,9,10], and, as a consequence, leads to a decrease in structural integrity. As mentioned by Djukic et al. [10], hydrogen embrittlement is related to the premature failure of metallic elements due to microstructure degradation and the appearance of characteristic cracks. A similar influence has the stress corrosion cracking, which was mentioned by Woodtli and Kieselbach in [8]. Various types of corrosion may attack metallic aircraft structures. These types vary depending on electrochemical and other physical processes being a driving force of corrosion, and, thus, different character and intensity. The most widespread types of corrosion include uniform and pitting corrosion, crevice corrosion, galvanic corrosion, microbiological corrosion, erosion, fretting, dealloying, exfoliation, already mentioned corrosion fatigue, and others. Details on the particular types mentioned above can be found in handbooks, see e.g., [7,11,12,13]. Among the numerous types of corrosion that occur in aircraft structures reviewed in [2], one of the special types of corrosion is hidden corrosion, which causes the so-called corrosion pillowing effect [2,14]. This type of corrosion was discussed in detail in the aforementioned publications in light of its appearance in elements of the aircraft fuselage, e.g., lap joints [15] and other airframe structures [16]. This type of corrosion occurs between at least two sheets of material joined by rivets. The pillowing effect causes barely visible local bulging of the material in the vicinity of joints, such as rivets, connecting the skins of a fuselage. Due to the breakdown of sealant or adhesive protection, moisture penetrates the joint, initiating a corrosion process by gradual oxidation. As a consequence, the products of corrosion cause an expansion of the skins between the rivets, introducing deformations referred to as the pillowing effect [14]. The corrosion products occupy several times more volume than the volume of the corroded material, and, in addition, their Young’s modulus is incomparably greater than the Young’s modulus of the joined elements. Due to this, the corrosion products, due to their much higher rigidity compared to the material of the joined sheets, begin to push apart the sheets joined together, which causes the appearance of characteristic bulges on the surface. This type of corrosion is especially dangerous, as it is not visible on the surface and cannot be detected by visual inspection (which is why it is called hidden corrosion), which requires the application of advanced non-destructive testing (NDT) techniques for its detection.

To prevent intensive corrosion in aircraft elements, numerous corrosion resistant materials are used for the construction of aircraft elements, including special steel alloys (such as Monel^®^), aluminum alloys (such as 1100, 2025, 2219, 3003, 5052) alloys and titanium alloys, and polymer matrix composites [13]. However, numerous aircraft with metallic airframes susceptible to corrosion are still in operation, and routine inspections of them using NDT techniques to detect corrosion spots are necessary to take appropriate maintenance steps.

For the inspection of corroded aircraft elements using NDT techniques, a variety of approaches are used. From the fundamental methods, usually penetrant testing or magnetic particle testing is used [13]. The most often used advanced NDT techniques are ultrasonic testing, eddy current testing, infrared thermography, radiography, and optical methods. Several examples of the evaluation of defects in riveted joints using the eddy current technique and X-ray computed tomography can be found in [17,18]. The usage of the latter techniques is defined by their high sensitivity to corrosion as well as their high probability of detection and quantification of corrosion [19]. A comparative study presented in [20] shows that in aircraft maintenance practice, many of the mentioned methods are applied simultaneously or in various combinations. Nevertheless, to reduce the costs and duration of inspections, some NDT techniques are favorable.

One of the effective NDT techniques for corrosion detection within the group of optical methods is the double pass retroreflection or D-Sight technique, which is based on observation of a tested structure at an oblique angle to detect mentioned deformations caused by corrosion. This technique was developed by Diffracto Ltd. in 1983 in Canada and later in 1988 implemented in Canada’s IAR NRC (Institute for Aerospace Research, National Research Council) for inspection of aircraft structures. The main advantage of this technique in comparison to other NDT techniques used for corrosion evaluation is the possibility of performing a low-cost and fast inspection for large areas with high sensitivity to surface deformations. Due to this, it found application in various inspection problems, but primarily in the inspection of automotive elements and aircraft fuselage structures for corrosion and barely visible impact damage [21,22,23]. For example, Hegeniers [24] described the application of the D-Sight technique to large area aircraft inspection, while Reynolds et al. [25] demonstrated an application of this method for sheet metal elements of automobiles and aircraft, as well as discussed the possibility of application of this method also to plastic and composite elements. Over the last three decades, the D-Sight technique has been improved mainly by its discoverers to adjust it for evaluation of corrosion and other types of damage in aircraft structures. For example, they proposed an approach based on numerical modeling of corrosion to support the quantification of hidden corrosion detected with the D-Sight technique. The results presented in [15] allowed an understanding of the character and distribution of stresses resulting from pillowing effect as well as defining the quantitative performance of the D-Sight technique. Further studies of this group [16,22] made it possible to develop appropriate quantitative metrics to estimate the residual life of aircraft structures. In addition, a similar approach to improving the D-Sight technique was described in [26]. In the last decade, little attention has been paid to improving the D-Sight technique, while recently several studies have been carried out on the improvement of the detectability of corrosion from D-Sight images with the support of artificial intelligence (AI) methods. In [19], the authors proposed an automated corrosion detection method in aircraft structures using deep neural networks and demonstrated great precision in corrosion detection using this method. In parallel, the authors of [27,28] demonstrated automatic corrosion detection from D-Sight images using deep learning and multi-teacher knowledge distillation.

However, the problem of evaluating the influence of corrosion in aircraft structures based on D-Sight images remains open. Regardless of the above-mentioned attempts to quantify corrosion, its applicability in inspection practice is not high because of numerous factors connected with uncertainties and incompleteness of experimental data obtained from D-Sight inspections. In this study, the authors proposed a new approach to the analysis of D-Sight inspection data, which is purely focused on the evaluation of collected images. From the practical point of view, it is essential to monitor the corrosion growth between performed inspections, and therefore, a method for analysis of historical inspection data is desired. However, since inspections are performed under variable conditions, such historical data are usually biased by uncertainties, such as different angles of observation or illumination. The authors proposed a method that eliminates these uncertainties and allows for monitoring corrosion growth based on a sequence of historical D-Sight images. To the best of our knowledge, this is the first attempt at the development of a tool for monitoring corrosion growth based on analysis of historical data obtained from D-Sight inspections. The performance of the method was demonstrated on inspection results of the Polish Air Force military aircraft obtained using the D-Sight NDT technique.

## 2. Materials and Methods

### 2.1. D-Sight Technique

**Figure 1:**
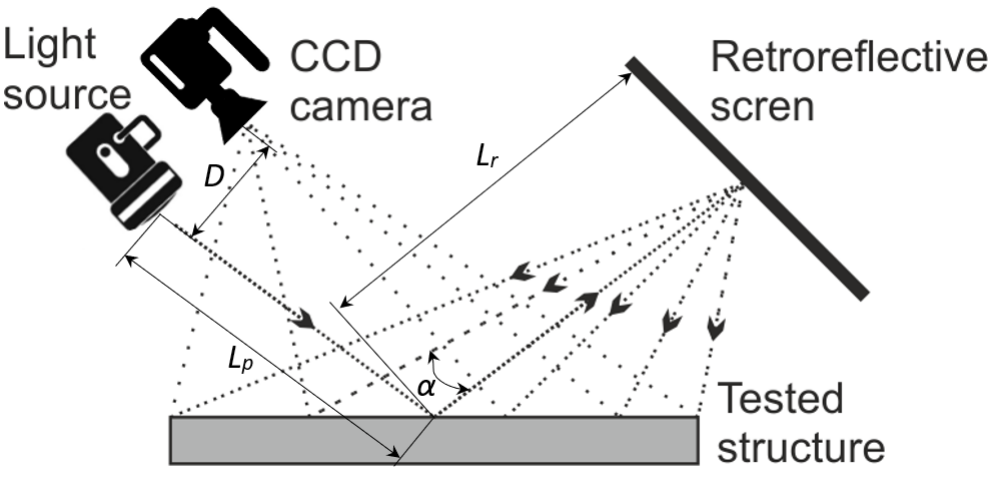
The scheme showing the principle of operation of the D-Sight technique [13].

The data acquisition system using the D-Sight phenomenon consists of a light source and a camera placed over the source (Figure 1). The distances from the camera to the point on a surface $L_{p}$ and from this point to the retroreflective screen $L_{r}$ presented in the figure are the paths of incidence and reflection of the light rays. It is obvious that the surface to be examined has an adequate light reflectivity coefficient. The reflectivity of the surface can be increased by using appropriate highlighters applied at the surface preparation stage. The light reflected from the surface backpropagate due to the retroreflective screen (mirror) presence. The screen is constructed from a grid of small objects of spherical geometry (the size of a single element is 60 micrometers) [29]. Thus, the constructed screen reflects the incident light rays at the same angle; however, its structure causes the reflected light rays to arrange themselves in a conical shape around the line of incidence (Figure 1). It is this basic feature of the phenomenon that made it possible to obtain D-Sight images. When the surface of the object under study is flat, the digital image captured by the camera has a uniform distribution of light intensity. If there are irregularities or deformations of the surface under inspection, the image collected by the camera will have disturbances in the distribution of light intensity (brightness contrasts). This is because the shape of the retroreflective screen causes a disturbance in the reflection of rays from the tested surface. The results are images in which the surface deformation can be seen as a bright area around the distortion, visible on one side, and a dark area on the other side of the distortion.

To fully understand the nature of the phenomenon, a mathematical description was introduced to establish the relationship between the various parameters of the collected data. The basic equation that describes the D-Sight phenomenon is one that determines the relationship between the angle of view $\alpha_{1}$ for the reflected light and the curvature of the surface under examination Δα [29]:

(1) $$\alpha_{1}=\left|\alpha_{0}+\Delta \alpha \right|\phi =\frac{D/\left(2\Delta \phi L_{p}\right)}{L_{p}+L_{r}},$$

where $\alpha_{0}$ is the incident angle for a flat or curved object ($\alpha_{0}=D/\left(L_{p}+L_{r}\right)$); Δα is the incident angle gradient by the difference between the local curvature of the surface and the curvature of the surface of the surrounding area; *D* is the distance between the position of the light source and the position of the recording camera.

Equation (1) allows one to determine the brightness level of the test area for the recorded image [29]:

- the brightness of the observed area will be less than the brightness of the surrounding area for Δα>0 and for $\Delta \alpha <-2\alpha_{0}$ (or Δϕ>0 and $\Delta \phi <-D/L_{p}$);
- the brightness of the observed area will be greater than the brightness of the surrounding area for $2\alpha_{0}<\Delta \alpha <0$ (or $-D/L_{p}<\Delta \phi <0$);
- maximum brightness will occur for $\Delta \alpha =\alpha_{0}$ (or $\Delta \phi =-0.5D/L_{p}$).

### 2.2. Inspected Structures and Experimental Setup

Based on the operation principle of the D-Sight technique, a commercial testing system called DAIS (D-Sight Aircraft Inspection System) was developed. The sensor consists of a digital camera and a white light source. The procedure for data acquisition with the DAIS system is to acquire digital images of the surface under test using a sensor connected to the acquisition unit (personal computer). The digital images recorded during the test are stored in a corresponding database. The database is connected to a schematic (data collection diagram) of the tested element. The diagram enables one to unambiguously assign the obtained image to a specific location in the examined element. Evaluation of the results of the test involves visual assessment and assignment of a specific type and intensity of damage. A digital image taken with DAIS has a resolution of 8 bits (grayscale mode). The data analysis of the images acquired during the inspection is evaluated based on expert experience. Data can be compared and assessed mostly qualitatively (an initial quantitative analysis using analysis of one-dimensional profiles was proposed in [25,30]).

**Figure 2:**
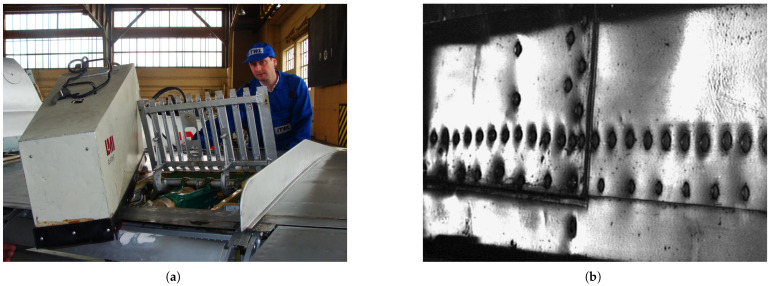
DAIS 250C during inspection of an aircraft (**a**) and exemplary D-Sight image of an aircraft skin panel (**b**).

This study is based on the results of routine NDT inspections of the skins of military aircraft of the Polish Armed Forces performed in the period of 2002 to 2017 using the D-Sight technique, which contains riveted connections. Previous unpublished analyzes of the acquired D-Sight images indicated the development of hidden corrosion of pillowing type in the vicinity of the rivets, and based on these analyzes, the exemplary cases were selected for further investigation in this study. The structures considered in this study were manufactured from aircraft aluminum alloys and contained paint on their surfaces. The inspections were performed using a DAIS 250C scanner system (Diffracto Ltd., Windsor, ON, USA) with a field of view of 580×131 mm and a resolution of the D-Sight image of 640×480 pixels. The scanning process is shown in Figure 2a, while the exemplary D-Sight image in Figure 2b.

## 3. Processing Procedure of D-Sight Images

**Figure 3:**
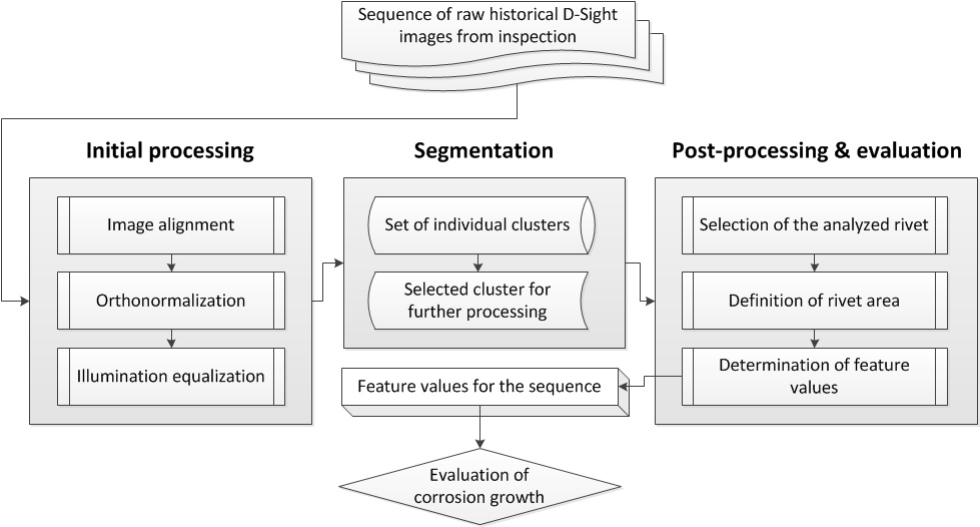
The flowchart of the applied processing algorithm of a sequence of D-Sight images.

As mentioned before, the D-Sight technique is used in practice mainly as a qualitative technique, which makes it possible to detect and localize spots of hidden corrosion and subjectively assess its severity. However, it is of great interest to monitor corrosion growth over the years of inspection, which is a challenging task when a great number of D-Sight image sequences need to be manually analyzed by inspectors. To improve this process, appropriate processing of D-Sight images is necessary. The initial concept of processing was communicated in [31]. The idea of monitoring corrosion growth in structures is based on the evaluation of historical D-Sight images collected during routine inspections of aircraft fuselage structures for a certain time period, which are processed using the developed image processing procedure. The general idea of the processing algorithm is presented in Figure 3, while the detailed description is presented in the next part of the following section.

### 3.1. Initial Processing

**Figure 4:**
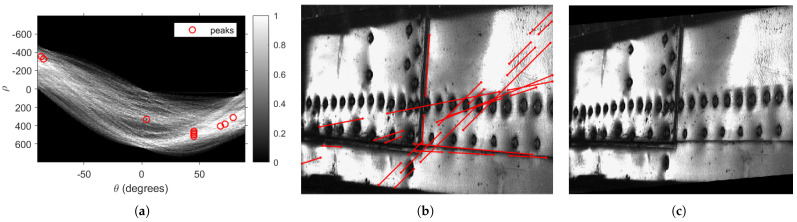
Image alignment: Hough transform matrix with identified peaks (**a**), D-Sight image with determined lines in principal directions (**b**), and aligned D-Sight image (**c**).

**Figure 5:**
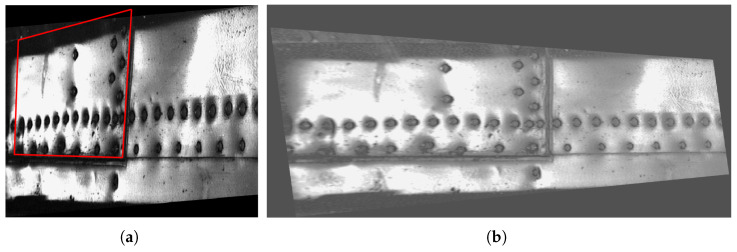
Orthonormalization: input image with the defined quadrangle (**a**), and the transformed D-Sight image with additional illumination equalization (**b**).

The inspections of aircraft elements using the D-Sight method are often performed in in-field conditions, where controlling numerous parameters of testing is very difficult or even impossible. According to this, collected historical data from inspections are usually affected by numerous factors, such as different angles of observation, resulting from variable curvatures of tested surfaces as well as operator’s mistakes, as well as inhomogeneous illumination from scan to scan. This makes particular D-Sight images within a historical sequence practically incomparable in terms of quantitative analysis. To overcome this problem, the initial processing procedure for D-Sight images was developed. The processing algorithms consist of three main steps:

1. Image alignment—at the first stage of processing, it was necessary to align the D-Sight image to make the selected edge visible on the image parallel to one of the edges of the image. This was necessary for a proper definition of reference points in the next steps. Within the alignment procedure, the Canny method was applied to perform edge detection on the D-Sight image, to which the Hough transform was applied to find lines in principal directions and determine their slopes. To determine these lines, 10 peaks were located in the Hough transform matrix with a threshold of 25% of the maximal value in this matrix (see Figure 4a). Knowing the angles, the D-Sight image was aligned using the shearing transform. The image with the determined lines and after shearing transformation is presented in Figure 4b and Figure 4c, respectively.
2. Orthonormalization—since the D-Sight images are always oriented with a specific angle into *z*-direction (due to the nature of the D-Sight testing technique—see Section 2.2), to make them comparable within a sequence, it was necessary to perform its orthonormalization to obtain its planar projection. To achieve this, it was necessary to input four reference points that form a quadrangle on an inspected surface for every D-Sight image (Figure 5a) and then used to compute the projective transformation matrix. Next, after performing a geometric transformation with the computed transformation matrix, one obtained the transformed D-Sight image (Figure 5b).
3. Illumination equalization—to neutralize the influence of illumination, the histogram equalization procedure was used. This procedure is based on mapping the gray levels of the initial image to the transformed image by using a transformation that operates on cumulative histograms and intensities of particular histogram components. A detailed description of the algorithm can be found e.g., in [32]. This operation performs contrast enhancement of a D-Sight image, making corrosion spots better visible. The final result of the initial processing of the exemplary D-Sight image is presented in Figure 5b.

The sequences of D-Sight images processed according to the algorithm presented above were then used as input to the segmentation algorithm specially developed to segment the corrosion spots and perform their quantitative analysis.

### 3.2. Segmentation

Image segmentation aims to group similar segments of an image with their respective cluster labels. In this study, grayscale image segmentation is performed using the univariate Gumbel finite mixture model and the Bayes decision theory. Here, the gray color of the pixel represents the random variable *y*, where y∈[0,…,255]. The three-parameter Gumbel parametric family implemented in [33] has been shown to be a good choice, since it prevents the accumulation of components at 0 and 255. Other distributions have problems at the interval boundaries and unjustifiably accumulate components there. Furthermore, the image of size n=width×height, where width and height determine the image size in pixels can be understood as a dataset $y_{1},\ldots ,y_{n}$ containing gray colors as integers. All calculations in this section were performed using the **rebmix** R package available at https://CRAN.R-project.org/package=rebmix, accessed on 29 September 2022, which was extended to address image segmentation in real time.

High-resolution images require long processing times. It can even be time-consuming to read them into the memory for further processing. Therefore, it is advisable to condense the image information into a histogram before segmentation. For this purpose, we have developed the method fhistogram. It outputs the histogram Hist object, which contains colors in the range from 0 to 255. To obtain an unbiased count of the pixel colors in the histogram, one must take $y_{\min}=-0.5$, $y_{\max}=255.5$, and the total number of bins K=256.

Hist <− fhistogram(Dataset = Image, K = 256, ymin = −0.5, ymax = 255.5,
  shrink = TRUE)
		
If one sets shrink to TRUE, each grayscale image is compressed to the maximum size of 2068 bytes for the float data type. This results in immense savings in memory usage and computation time. Looking at histograms, one can also identify potential flaws in the images. In the study, we observed histogram peaks in the darkest color and the lightest color or only in the darkest color. Some images were also contained in a black frame with a thickness of one pixel. These flaws were filtered out initially.

For illustration, the initially selected exemplary D-Sight image (Figure 2b) was used for further processing. When the code section

EM <− new("EM.Control", strategy = "exhaustive")
rebmixest <- REBMIX(Dataset = list(Image), Preprocessing = "histogram",
  cmax = 30, Criterion = "BIC", pdf = "Gumbel", theta3 = NA,
  ymin = −0.5, ymax = 255.5, K = 256, EMcontrol = EM)
		  
is executed, the Image of size *n* = 3,041,906 is processed by the REBMIX&EM algorithms [34,35]. If in the code section EMcontrol is set to NULL, the image will be processed only by the REBMIX algorithm. If instead, the code section

rebmixest <- REBMIX(Dataset = list(Hist), cmax = 30, Criterion = "BIC",
  pdf = "Gumbel", theta3 = NA, EMcontrol = EM)
		
is executed, the histogram will be processed by the REBMIX&EM algorithms. It turns out that the third option is 9790 times faster than the first and 2422 times faster than the second. The third option is even four times faster than simply reading the image into memory. In terms of accuracy, the first and second options yield the same Bayesian information criterion BIC = 25,909,362 at c=28 components, while the third option yields a better value BIC = 25,907,789 at the same number of c=29 components. Thus, the third option proves to be the best and therefore be used further on for image processing.

In D-Sight images, some colors are missing in the gray levels between 0 and 255, which is due to the histogram equalization operation performed during initial processing (see Section 2.1 for details). Thus, it is not mandatory that K=256 is optimal. The optimal number of bins is the one that leads to the minimum BIC. The method fhistogram is run for *K* one of 256, 128, 64, and 32 as shown above, and the parameters of the finite Gumbel mixture model are estimated by calling the method REBMIX. Thus, for K = 256, 128, 64, and 32, we get BIC = 13,869,903 at c=29, BIC = 14,830,797 at c=30, BIC = 16,539,215 at c=27 and BIC = 16,480,251 at c=18, respectively. K=256 seems, therefore, to be optimal and will be used in the remainder of this paper.

The next step in image segmentation is clustering. In the package **rebmix** we have the method RCLRMIX. Previously, it was called as

rebmixclr <- RCLRMIX(x = rebmixest, Dataset = Image, Rule = "Entropy")
		
The method performs cluster merging based on various rules. For the purposes of this study, the entropy rule developed in [36] was used. It was found that the method is very time consuming for image processing. Therefore, it was extended for histogram input. When code section

rebmixclr <- RCLRMIX(x = rebmixest, Rule = "Entropy")
Zp <- mapclusters(x = rebmixclr, Dataset = Image)
 
is executed instead, the merging of components is done in RCLRMIX, and the clustering according to the Bayes decision theory is done in mapclusters. In this way, we obtain the same results 140 times faster. However, it must be emphasized that this part of the code takes 3.9 times more time than just reading the image into memory. This is entirely due to mapclusters, which still works with the image and not the histogram. RCLRMIX here is 44 times faster than reading the image.

**Figure 6:**
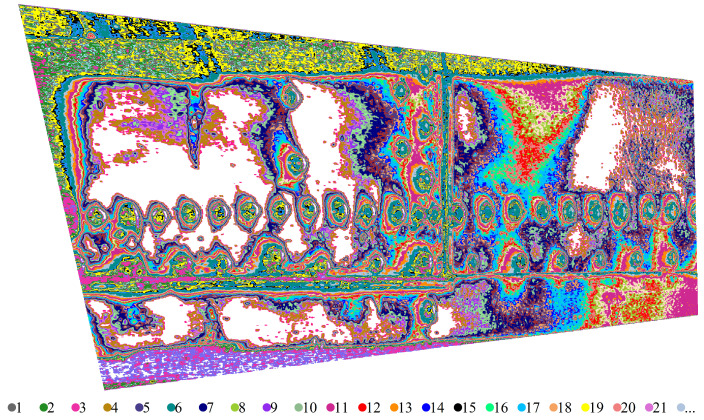
The example of clustering of a D-Sight image for K=256.

**Figure 7:**
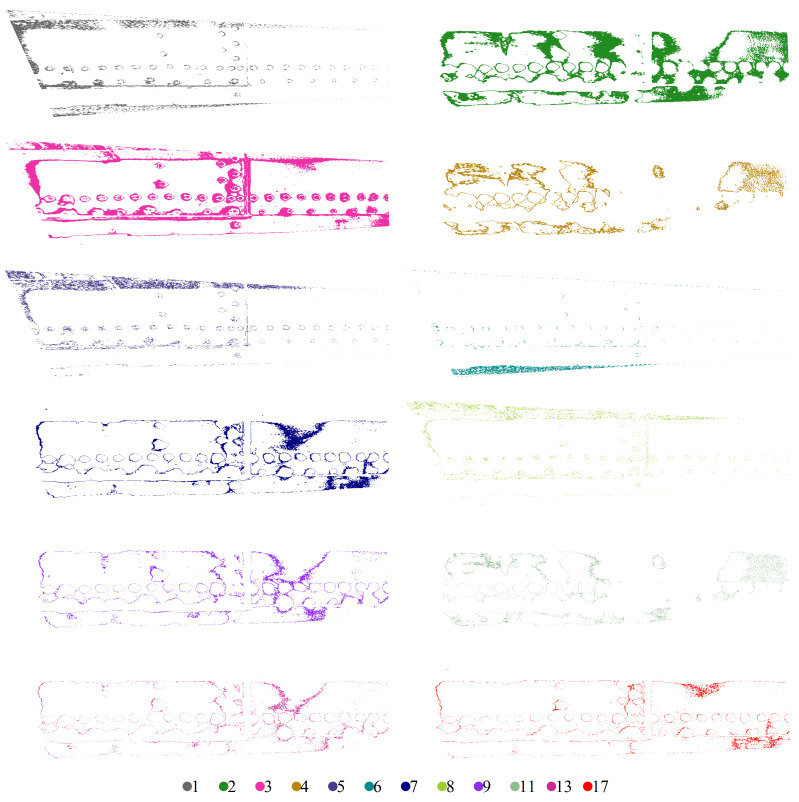
Individual clusters were extracted for an exemplary D-Sight image.

For K=256, the REBMIX yields 29 components. It turns out that clustering leads to 29 clusters, which are shown in Figure 6. It can be observed that there are too many similar clusters denoting the same image features. We, therefore, repeat the calculations for K=32, resulting in only 18 components. The clustered image consists of 12 clusters and is shown in Figure 7. Thus, the number of similar clusters was reduced. Therefore, K=32 was used to process all the images. We see that an optimal BIC does not necessarily mean optimal clustering. The entropy rule suggests that we merge the components from right to left of Table 1. This means that we first merge the component 9 with the component 2, then the component 10 with the component 2, and so on.

**Figure 8:**
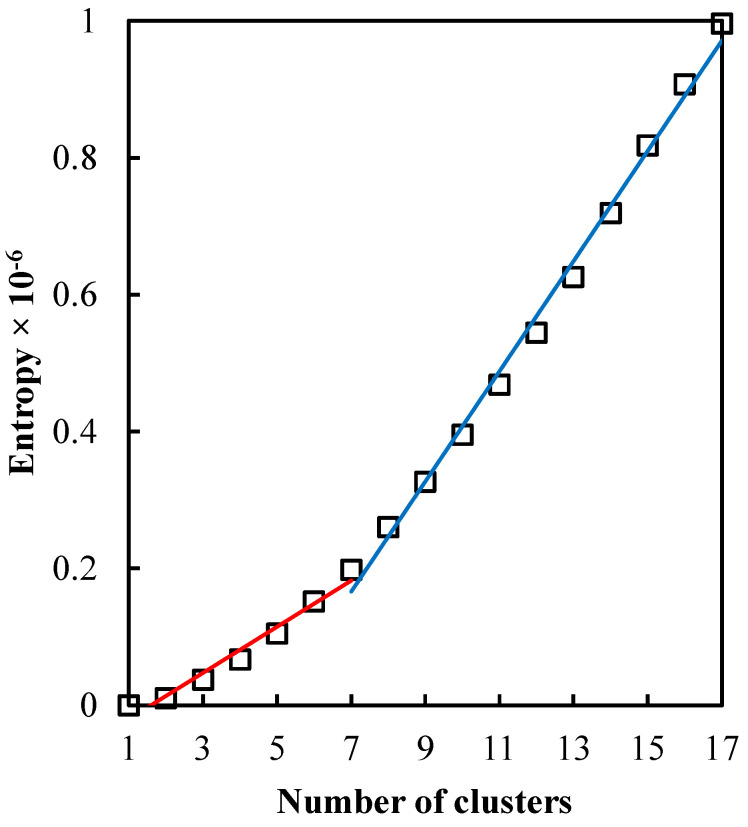
Entropy values for the *c*-cluster combined solution. The regression lines show the best piecewise linear fit, with a breakpoint at c=7 clusters.

When all components are merged, we get only one component labeled 1. It should be emphasized that not all cluster labels appear when clustering by mapclusters. The available clusters are indicated by underlining in Table 1. In [36], it is suggested that the optimal number of clusters coincides with the knee point in Figure 8. In our case, the knee point is barely visible with 7 clusters.

When manually examining the clustered image in Figure 7, one can observe two distinct groups of clusters. The first group consists of clusters with rivets (clusters 1, 3, 5, 6, and 8) and the second group (2, 4, 7, 9, 11, 13, and 17) consists of clusters without rivets. Thus, manual merging would indicate that the first group of clusters is important for identifying corrosion. The rest could be treated as background. Even the first group contains backgrounds that cannot be filtered out using the segmentation technique presented. The entropy rule proposes to merge the clusters 3 and 2, 8, and 2, and 2 and 1, which is in contradiction with the human decision. This means that the process of merging clusters in this particular case could not be automated yet.

### 3.3. Post-Processing and Evaluation of Corrosion Growth

Due to the applied orthonormalization in the pre-processing step, the resulting images change their dimensions. Moreover, the areas of observation, as well as the distances to the tested structure and the angle of observation are usually differ. According to this, it is essential to normalize the dimensions of the images to make them comparable. This dimensional normalization was performed based on the equalization of distances between selected pairs of rivets visible in the entire sequence of images.

In the last step of processing, a pre-processed and segmented D-Sight image is assessed in terms of the detection of corroded areas and evaluation of corrosion severity. For this purpose, a given rivet is extracted from the image and recorded in the form of a rectangular matrix with pixels representing grayscale values from 0 to 255. The accuracy of the representation of a rivet with a surrounding corroded area is determined by the resolution of the initial image, which, in turn, is connected with the resolution of a camera used in the measurement device (see Section 2.2 for more details).

**Figure 9:**
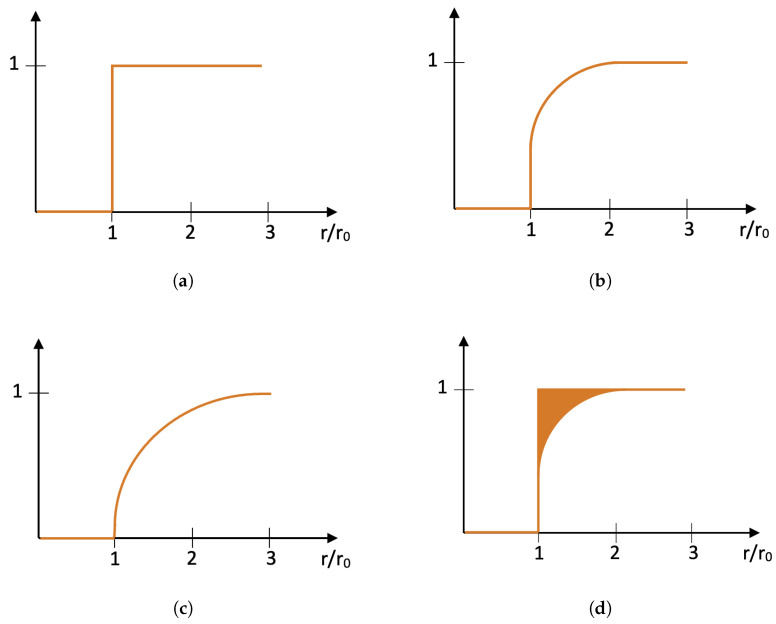
Exemplary schemes of a functional measure for evaluation of corrosion severity: (**a**) no corrosion, (**b**) light corrosion, (**c**) severe corrosion, and (**d**) graphical representation of a feature for evaluation of corrosion severity.

The rivet is identified by three parameters: the radius $r_{0}$ and the coordinates $(x_{0},y_{0}$ of its spatial location. For the rivet defined by these parameters, the concentric circles are determined with a step assumed as $0.05r_{0}$, which resulted in 59 values. The series determined in this way is a functional measure of corrosion severity, which is schematically shown in Figure 9 for various levels of corrosion severity. Based on the area above the curve, one can define the feature that describes the corrosion severity quantitatively (see Figure 9d).

**Figure 10:**
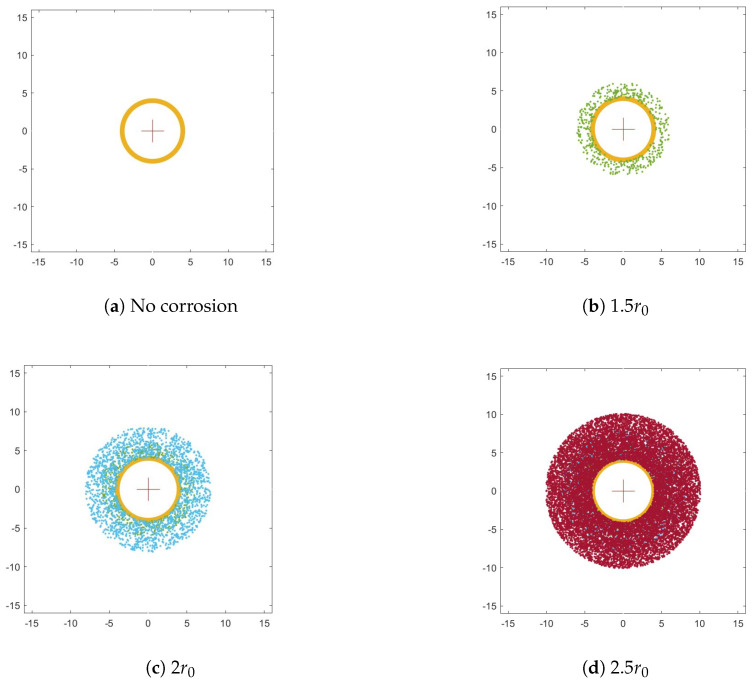
Simulated hidden corrosion around the rivet.

**Figure 11:**
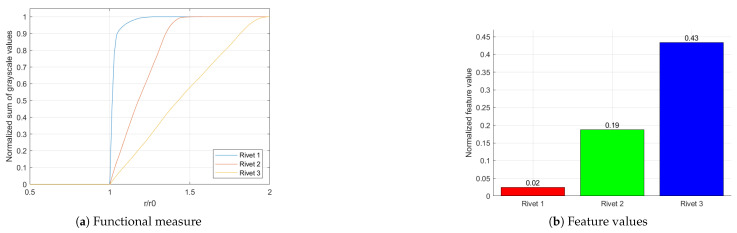
The results of the corrosion growth evaluation for simulated hidden corrosion.

In addition to evaluating the severity of the corrosion, it was necessary to develop a tool with the possibility to evaluate the growth of the corrosion according to the objectives of this study. For this purpose, the initial tests of the proposed approach were performed on artificially generated images simulating hidden corrosion (see Figure 10). These images represent the process of corrosion growth and consider not only the increase in its area but also its intensification, which is simulated by a power function. This explains the non-linear character of the resulting curves; however, as presented in the next sections, it properly reflects the character of distribution of hidden corrosion around a rivet. The procedure described above was applied to the simulated images to determine functional measures and corresponding feature values that represent corrosion growth. The results obtained for the simulated images are presented in Figure 11. The presented results clearly show the performance of the applied algorithm in the quantitative evaluation of corrosion growth.

## 4. Demonstration of the Processing Algorithm Performance

**Figure 12:**
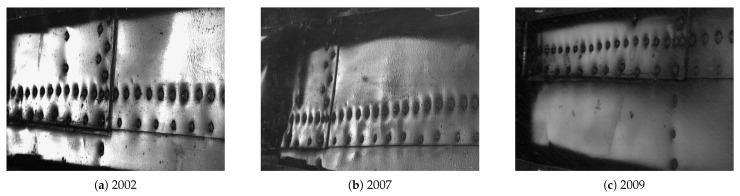
Exemplary D-Sight images of the same riveted aircraft panel inspected in various years.

In the next step, the algorithm was applied to the sequence of D-Sight images, which represent the same aircraft panel tested with the D-Sight method during its operation in periodical inspections, see Figure 12. In the presented example, one can clearly see the mentioned challenges in the processing of D-Sight images from periodic inspections. In particular, one can observe that the images reveal differences in the captured areas as well as in the angle of observation and illumination, which justifies the requirement of an application of the above-described procedures before quantitative evaluation.

**Figure 13:**
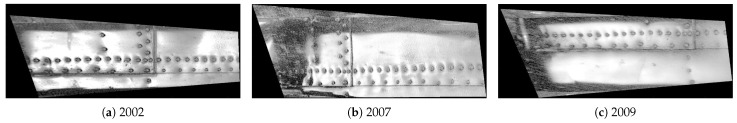
The D-Sight images after pre-processing.

**Figure 14:**
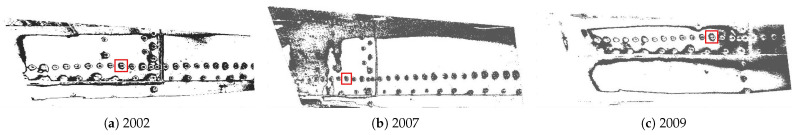
The D-Sight images after segmentation with an indication of the analyzed rivet.

The selected D-Sight images were firstly pre-processed and segmented according to the procedures described in Section 3.1 and Section 3.2. The results of these steps are presented in Figure 13 and Figure 14, respectively. It should be noted that the results presented in Figure 14 represent only one cluster selected for further processing: in the first case, it was cluster no. 3, while in the rest cases, it was cluster no. 1.

**Figure 15:**
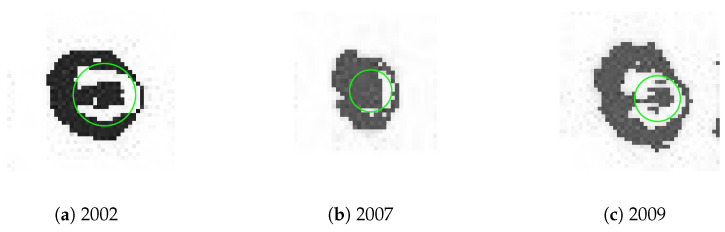
The extracted grayscale images of the selected rivet.

**Figure 16:**
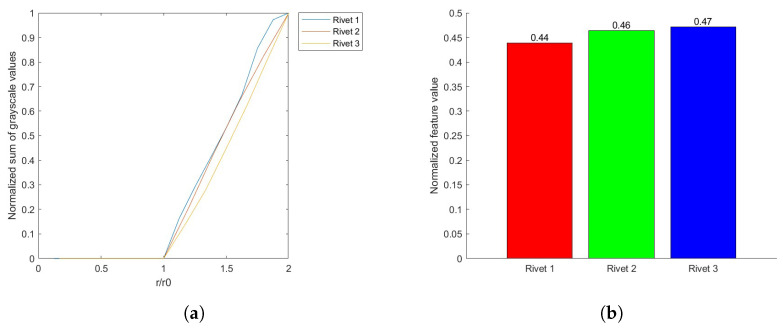
The functional measure (**a**) and feature values (**b**) obtained for the analyzed rivet.

The evaluation of the corrosion growth was then performed for the selected rivet marked with red squares in Figure 14. The processed and extracted images of the selected rivet are presented in Figure 15, while the results obtained for this rivet are presented in Figure 16. This example shows that the corrosion grows continuously from 2002 to 2009. It is worth mentioning that the presented example is not representative of the corrosion growth process, i.e., it can grow in a non-monotonic way depending on the numerous operation and environmental factors and may differ significantly even for rivets located close to each other. According to this, the case studies for other sequences of D-Sight images are presented and discussed in the next section.

## 5. Discussion

To demonstrate the performance of the developed algorithm, the selected case studies are presented in Appendix A, which demonstrate the evaluation of corrosion growth over time in aircraft panels for selected rivets. These case studies were also selected to support the discussion provided in this section.

The performed case studies in Appendix A show the performance of the proposed processing method and the ability of quantitative evaluation of the corrosion growth based on D-Sight images acquired from aircraft structures in typical inspection conditions. This exposed numerous difficulties and challenges in processing D-Sight images and estimation of the corrosion growth, which was resolved in particular procedures described in Section 3. The initial goal of this study was fulfilled; however, the obtained results draw new problems and challenges in processing D-Sight images, which are discussed in this section.

**Figure A1:**
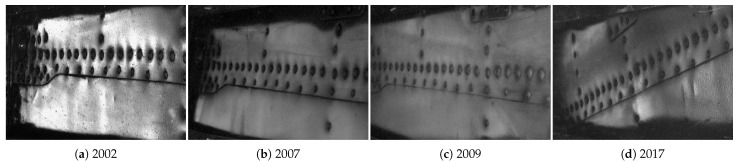
Case 1: the sequence of D-Sight images of the analyzed aircraft panel.

The primary source of possible errors and inaccuracies in acquired D-Sight images is the variability of the inspection conditions mentioned above, that is, the area and angle of observation and illumination during the inspection. Considering that NDT inspections using the D-Sight technique are usually performed in hangars or in field conditions as well as repeating inspections are performed in intervals of several years, it is difficult to ensure conditions close to laboratory conditions. The developed algorithms described in this paper successfully limit the rate of possible errors; however, one should consider that their full elimination might not be possible. Moreover, due to differences in the area of observation, i.e., spatial shifts in sequences of D-Sight images over time (see e.g., sequences presented in Figure 12, Figure A1 and Figure A5), the evaluation of corrosion growth is possible only for those locations, which are well observable in all D-Sight images in the sequence. Finally, the accuracy of the estimation of corrosion growth depends on the resolution of the D-Sight images, which can be assessed as quite low considering the characteristics of cameras currently available in the market. Moreover, low resolution might be a source of additional noise and recognition of corrosion due to illumination deficiencies. Finally, the direction of illumination influences the process of evaluation of hidden corrosion around rivets, as the corrosion can be distributed in a non-uniform character around the rivet and, due to illumination, inhomogeneity might be less visible. Nevertheless, as shown in the presented study, the resolution of D-Sight images is enough to evaluate corrosion growth over intervals of several years and is consistent with requirements in the inspection of aircraft structures.

**Figure A4:**
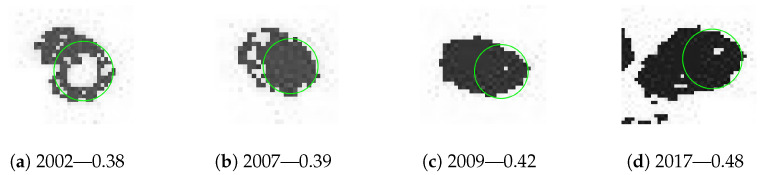
Case 1: the extracted grayscale images for the selected rivet with feature values obtained for this rivet for subsequent years of inspection.

**Figure A5:**
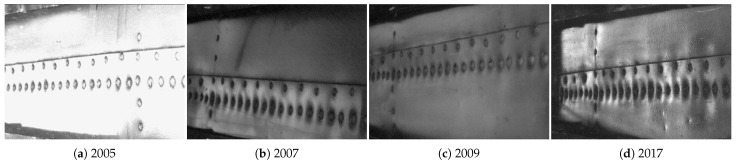
Case 2: the sequence of D-Sight images of the analyzed aircraft panel.

**Figure A7:**
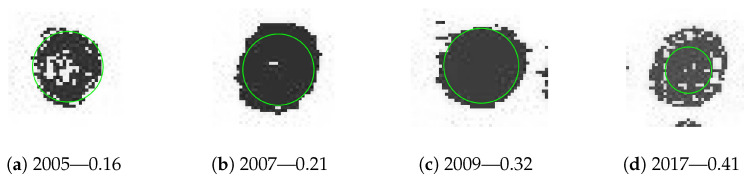
Case 2: the extracted grayscale images for the selected rivet with the feature values obtained for this rivet for subsequent years of inspection.

**Figure A10:**
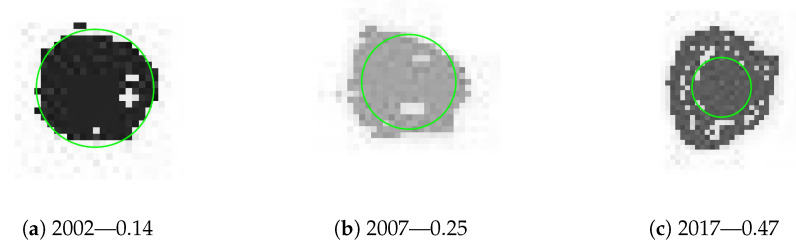
Case 3: the extracted grayscale images for the selected rivet with the feature values obtained for this rivet for subsequent years of inspection.

**Figure A13:**
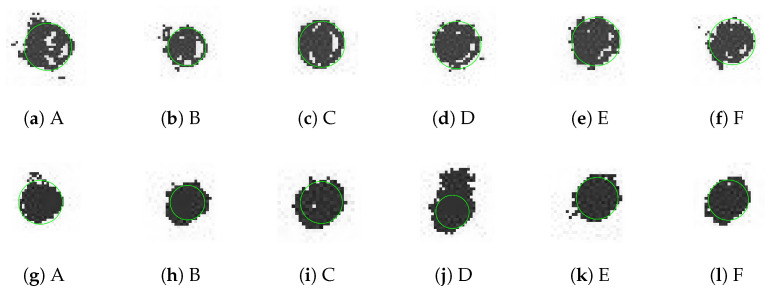
Case 4: the extracted grayscale images for the selected rivets during inspection in 2002 (**a**–**f**) and in 2017 (**g**–**l**).

The secondary source of possible errors is the processing algorithm. Due to the differences in the angle of observation and illumination for particular D-Sight images in a sequence, the initial processing, which includes alignment, orthonormalization, and illumination equalization, is necessary to make the images comparable. During the first two substeps, the proportions of the resulting images are not preserved; however, this problem is resolved at the final processing step by selecting the boundaries of a rivet (see Section 3.3). The pre-processing procedures might generate some additional errors due to geometric and color transforms applied in this step. Next, the applied segmentation algorithm, although very effective, is based on the manual selection of an appropriate cluster for further processing. The well-tuned segmentation algorithm allows for minimizing errors at this step; however, minor errors, in particular, in the estimation of the shape and area of a given rivet surrounded by corroded area, may also appear during this procedure. Due to the already mentioned variability in the input data, the automation of this step is a great challenge. At the post-processing step, the estimation of the boundaries of a given rivet, as well as evaluation of the corroded area, is also limited by image resolution, which can be visible on numerous magnifications of rivets presented in this study (see Figure 15, Figure A4, Figure A7, Figure A10 and Figure A13). Nevertheless, the evaluation of corrosion growth in the intervals between inspections is possible to assess.

Despite the challenges discussed above for the quantitative evaluation of corrosion growth, the developed processing method can be considered an effective support tool to analyze the progress of structural damage in aircraft elements subjected to hidden corrosion, which might be used in the planning of further maintenance, including repairs and replacements of particular elements.

## 6. Conclusions

The presented study focused on improving the estimation of the growth of hidden corrosion, known as pillowing corrosion, in metallic aircraft structures by developing and applying the processing method, which made it possible to evaluate the growth of corrosion quantitatively. The developed method based on geometric transforms and an advanced segmentation algorithm is the first successful implementation of quantitative evaluation of hidden corrosion growth in aircraft structures based on NDT results obtained using the D-Sight technique and can be considered as a first step in the D-Sight-based structural health monitoring (SHM) technique. The developed method was tested on D-Sight images obtained during the inspection of military aircraft structures of the Polish Armed Forces and its performance was justified in the presented case studies. These studies allowed defining open problems and challenges in the monitoring of hidden corrosion based on analysis of D-Sight images and directions of further development of this approach. This is a part of ongoing studies that led to the development of fully quantitative NDT and SHM techniques based on the classical D-Sight technique.

The proposed processing algorithm has some limitations, discussed in detail in Section 5, which include uncertainties appearing directly from the inspection conditions and possible errors resulting from the processing of D-Sight images. In the first case, the uncertainties cannot be fully removed since in maintenance practice, especially in the case of military aircraft, inspection is often performed in field conditions; thus, the angles of view and illumination cannot be controlled as in the laboratory conditions. The developed pre-processing procedures demonstrated good results in solving this problem; however, further studies on the improvement of these algorithms are planned. The errors resulting from the processing algorithm are mainly to the appropriate evaluation of a zone in the vicinity of a rivet affected by hidden corrosion. Although the performance of the developed algorithm demonstrated satisfactory results, there are still unsolved issues, such as the necessity of manual selection of clusters in the segmentation step, as well as the definition of a diameter of an analyzed rivet in the last processing and evaluation step. In further studies, it is planned to automate these routines to make the algorithm developed easier to use as an inspection support.

## Figures and Tables

**Table 1:** Merging of components according to the entropy rule. The check marks indicate agreement or disagreement with the manual merging.

*c*	1	2	3	4	5	6	7	8	9	10	11	12	13	14	15	16	17
from	16	12	6	15	2	8	4	11	5	14	18	17	13	7	3	10	9
to	1	1	1	1	1	2	2	2	1	2	2	2	2	2	2	2	2
check			✓		✗	✗	✓	✓	✓			✓	✓	✓	✗		✓

**Figure A2:**
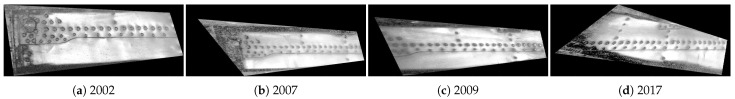
Case 1: pre-processed D-Sight images of the analyzed aircraft panel.

**Figure A3:**
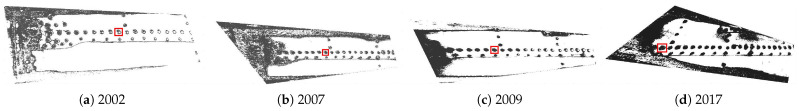
Case 1: selected clusters from segmentation of D-Sight images of the analyzed aircraft panel.

**Figure A6:**
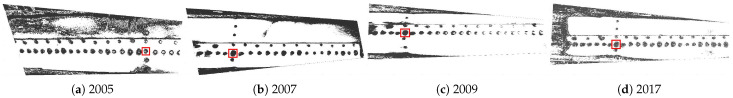
Case 2: selected clusters from the segmentation of D-Sight images of the analyzed aircraft panel.

**Figure A8:**
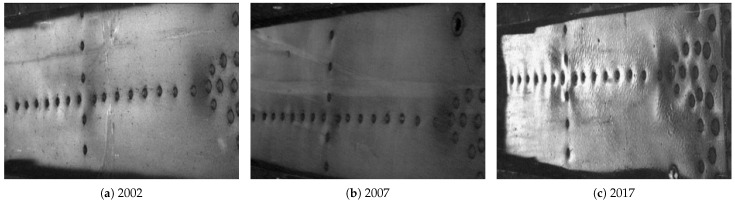
Case 3: the sequence of D-Sight images of the analyzed aircraft panel.

**Figure A9:**
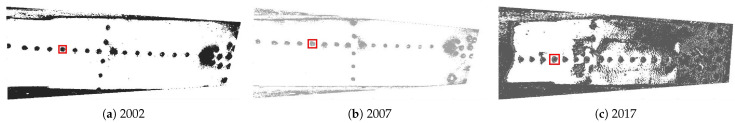
Case 3: selected clusters from the segmentation of D-Sight images of the analyzed aircraft panel.

**Figure A11:**
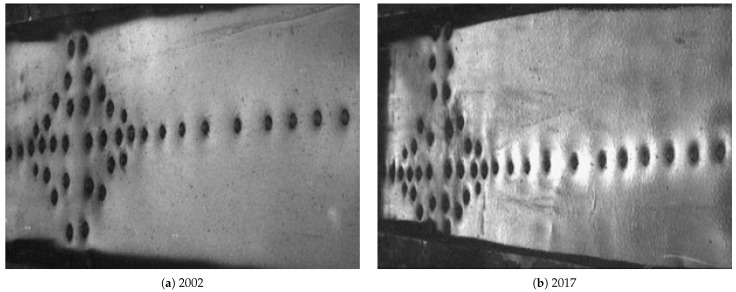
Case 4: the sequence of D-Sight images of the analyzed aircraft panel.

**Figure A12:**
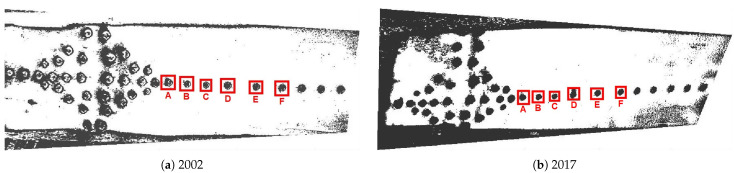
Case 4: selected clusters from the segmentation of D-Sight images of the analyzed aircraft panel.

**Figure A14:**
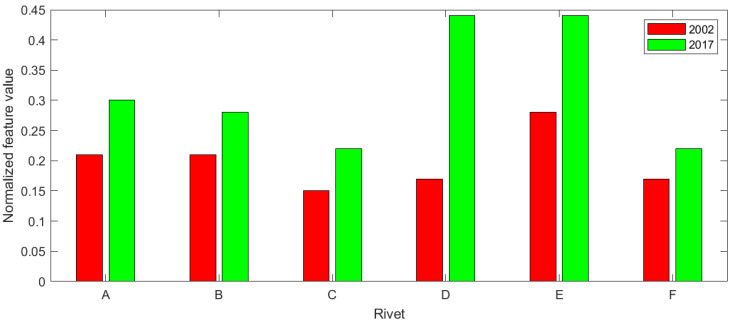
Case 4: the feature values obtained for the analyzed rivets.

## Data Availability

Restrictions apply to the availability of these data. Data were obtained from the Air Force Institute of Technology in Warsaw and are available from the corresponding author with the permission of the Air Force Institute of Technology in Warsaw. The REBMIX software developed and improved for segmentation is available on https://cran.r-project.org/web/packages/rebmix/index.html, (accessed on 29 September 2022).

## Appendix A. Case Studies

### Appendix A.1. Case 1—Intersection of Rows of Rivets

In this study, we selected an aircraft panel with two horizontal rows of rivets, and the rivet selected for analysis is located close to the row of rivets perpendicular to the two mentioned rows. This selection is justified by visible corrosion in its surrounding, which can be a result of a higher probability of access of moisture to this riveted joint. The sequence of analyzed D-Sight images in this study is presented in Figure A1. The images were pre-processed and segmented according to the procedures presented in Section 3.1 and Section 3.2, and the results for these steps are presented in Figure A2 and Figure A3. The clusters from segmentation were appropriately selected to capture images with well-distinguishable rivets with corrosion. The rivet selected for the quantitative analysis was marked with red squares in the latter Figure. The rivets surrounded by corrosion and the resulting feature values for particular years of inspection are presented in Figure A4.

### Appendix A.2. Case 2—Intersection of Rows of Rivets

The sequence of D-Sight images for another case of a similar type is presented in Figure A5, which represents the aircraft panel inspected in 2005, 2007, 2009, and 2017 with the rivet subjected to corrosion growth at the intersection of horizontal and vertical rows of rivets. The corrosion growth is visible on raw D-Sight images, however, the analysis was performed to evaluate its growth quantitatively. After pre-processing and segmentation, the rivet of interest is selected and analyzed. The D-Sight images after segmentation and selected images of the rivet with the accompanying feature values are presented in Figure A6 and Figure A7, respectively. The obtained feature values confirm the visually observed corrosion growth. It can be noticed that the corrosion growth is much more intensive than for the rivet analyzed in Case 1. A significant increase is observed from 2009 to 2017, which is visible comparing both the images and feature values; see Figure A7c,d.

### Appendix A.3. Case 3—Rivet in a Row Far from the Intersection

The next case presents the corrosion growth near the rivet on the aircraft panel located far from other joints. The D-Sight images from the inspection for this case are presented in Figure A8. The corrosion growth is also well visible when one compares the D-Sight images. The quantification of this growth was performed for the rivet marked with red squares (see Figure A9) and the obtained results are presented in Figure A10. Despite the lower probability of corrosion growth at this location, in the 15-year period, the corroded area around the rivet increased more than three times.

### Appendix A.4. Case 3—Rivet in a Row Far from Intersection

The last case considered in this study represents the analysis of corrosion growth in the surrounding rivets on the aircraft panel in the 15-year time interval for selected multiple rivets located in one row. The analyzed D-Sight images for this case are presented in Figure A11. The corresponding pre-processed and segmented images with the indicated rivets considered in the analysis are shown in Figure A12, while the enlarged rivets with the marked rivet envelope are shown in Figure A13. It is clearly observable that the corrosion increased in the surrounding of the analyzed rivets both by visual analysis of raw D-Sight images and their segmented versions. The results of the quantitative evaluation for this case are presented in Figure A14. From the presented results, one can observe that the rate of corrosion growth is different for rivets located in the same row. This is especially well visible for rivet D, for which the increase was the most significant.
